# Increased gene expression and copy number of mutated *bla*_KPC_ lead to high-level ceftazidime/avibactam resistance in *Klebsiella pneumoniae*

**DOI:** 10.1186/s12866-021-02293-0

**Published:** 2021-08-19

**Authors:** Lingxiao Sun, Haibo Li, Qi Wang, Yingmei Liu, Bin Cao

**Affiliations:** 1 National Clinical Research Center for Respiratory Diseases, China-Japan Friendship Hospital, Capital Medical University, Beijing, China; 2 Laboratory of Clinical Microbiology and Infectious Diseases, Department of Pulmonary and Critical Care Medicine, Center for Respiratory Diseases, China-Japan Friendship Hospital, Beijing, China; 3 National Clinical Research Center of Respiratory Diseases, Institute of Respiratory Medicine, Chinese Academy of Medical Science, Beijing, China; 4 Department of Clinical Laboratory, Peking University People’s Hospital, Beijing, China; 5 Tsinghua University-Peking University Joint Center for Life Sciences, Beijing, China

**Keywords:** Ceftazidime/avibactam resistance, Minimal inhibitory concentration, Mutated *bla*_KPC_, Outer membrane protein, Electroporation

## Abstract

**Background:**

Resistance to ceftazidime-avibactam was reported**,** and it is important to investigate the mechanisms of ceftazidime/avibactam resistance in *K. pneumoniae* with mutations in *bla*_KPC_.

**Results:**

We report the mutated *bla*_KPC_ is not the only mechanism related to CZA resistance, and investigate the role of outer porin defects, efflux pump, and relative gene expression and copy number of *bla*_KPC_ and *ompk35/36*. Four ceftazidime/avibactam-sensitive isolates detected wild type *bla*_KPC-2_, while 4 ceftazidime/avibactam-resistant isolates detected mutated *bla*_KPC_ (*bla*_KPC-51_, *bla*_KPC-52_, and *bla*_KPC-33_). Compared with other ceftazidime/avibactam-resistant isolates with the minimal inhibitory concentration of ceftazidime/avibactam ranging 128–256 mg/L, the relative gene expression and copy number of *bla*_KPC_ was increased in the isolate which carried *bla*_KPC-51_ and also showed the highest minimal inhibitory concentration of ceftazidime/avibactam at 2048 mg/L. The truncated Ompk35 contributes rare to ceftazidime/avibactam resistance in our isolates. No significant difference in minimal inhibitory concentration of ceftazidime/avibactam was observed after the addition of PABN.

**Conclusions:**

Increased gene expression and copy number of mutated *bla*_KPC_ can cause high-level ceftazidime/avibactam resistance.

## Background

Carbapenem-resistant Enterobacerales (CRE), especially carbapenem-resistant *Klebsiella pneumonia* (CRKP) have emerged as a major public health concern worldwide. In China, the production of *K. pneumoniae* carbapenemases (KPCs) is the predominant mechanism of carbapenem resistance and is frequently linked to a highly successful *K. pneumoniae* sequence type 11(ST11) clone [1]. The existing antibiotics treating infections caused by KPC-producing *K. pneumoniae* (KPC-Kp) have limited efficacy, and novel antibiotics are urgently needed.

Avibactam is a non-β-lactam, β-lactamase inhibitor that inhibits the activities of Ambler class A and C β-lactamases and some Ambler class D enzymes. Ceftazidime/avibactam (CZA) has been considered a promising β-lactam-β-lactamase inhibitor combination with activity against serine β-lactamases, including KPCs [2]. However, CZA resistance has been reported in patients after short periods of CZA exposure [3–17], and also in patients with no history of CZA therapy [18–23]. Mechanisms of CZA resistance have reported in several studies, including specific mutations in the *bla*_KPC_ gene [4], specific mutations in the *bla*_CTX-M_ gene [17], porin deficiency combined with high ceftazidime hydrolysis [19, 20], or porin inactivation with increased expression of the *bla*_KPC_ gene [21, 22]. The mechanism most often associated with the emergence of CZA resistance after treatment has been observed to be mutations in the *bla*_KPC_ gene encoding for KPC enzymes [4–12]. And the most common amino acid substitution of KPC was D179Y in KPC-2 (KPC-33) [6] and KPC-3 (KPC-31) [4].

CZA has been approved by the China State Drug Administration on May 21, 2019, for the treatment of complex intra-abdominal infections (cIAI), hospital-acquired pneumonia (HAP), and for the treatment of gram-negative bacterial infections in adults with limited therapeutic options: *K. pneumoniae*, *E. cloacae*, *E. coli*, *Proteus mirabilis*, and *Pseudomonas aeruginosa*. Before the wide use of CZA, CZA-resistant isolates carrying wild type KPC-2 have been reported in China [19, 23]. Besides, we reported our experience in treating ten lung transplant recipients (9 with KPC-Kp infections, and 1 with *Pseudomonas aeruginosa* infection) with CZA at the China-Japan friendship hospital [24]. CZA resistant KPs with mutated *bla*_KPC_ were recovered from 4 patients after CZA treatment [25]. Plasmid transfer and *bla*_KPC_ cloning showed the mutated *bla*_KPC_ in these isolates were associated with CZA resistance. We observed that the minimal inhibitory concentration (MIC) for CZA of KPC-51-producing *K. pneumoniae* clinical isolate was the highest (2048 mg/L) in our study, while the CZA MIC for the KPC-51-harbouring *E. coli* DH5a transformant was only 8 mg/L. We believed that the mutated *bla*_KPC_ was only partly contributing to the resistance of CZA in this isolate, and other resistance mechanisms should be further investigated. Besides, though increased gene expression and copy number of *bla*_KPC_ and/or porins defects were reported associated with CZA resistance, this finding was often reported in isolates with wild type *bla*_KPC_, and rarely reported in isolates with mutated *bla*_KPC_. The role of *bla*_KPC_ expression and porins in CZA resistant isolates with mutated *bla*_KPC_ is not clear, especially in isolates with different resistant levels.

## Results

### Bacterial isolates

After 13–22 days of CZA treatment in lung transplant patients, CZA resistance was found in 4 isolates. These 4 CZA resistant isolates (1B, 3B, 7B, 8B) and 4 baseline isolates (1A, 3A, 7A, 8A) recovered before CZA treatment from the same patients were analyzed in the present study. All 8 isolates produced KPC, and other beta-lactamases were detected (Table 1). Four baseline isolates carrying wild type *bla*_KPC-2_ were susceptible to CZA, while the *bla*_KPC_ of corresponding CZA-resistant isolates were mutated after CZA treatment. KPC-33(D179Y), KPC-51(D179N, Y241H, H274N), and KPC-52(D179Y, valine insertion after 262 position) were observed in 8B, 1B, and 7B, respectively. Among the 1228 reads covering KPC in the next-generation sequencing data of isolate 3B, 349 reads (28.4%) belonged to KPC-2, and 879 reads (71.6%) belonged to KPC-33. The transformed *E.coli* isolates carrying mutated *bla*_KPC_ manifested increased CZA MICs compared with the WT *bla*_KPC-2_ transformant. The CZA MIC of 3B, 7B, and 8B were 256, 256, and 128 mg/L, and the CZA MIC in corresponding transformed *E. coli* isolates harboring the same *bla*_KPC_ variants were 2, 32, and 2 mg/L, respectively [25]. It is worth noting that the CZA MIC of KPC-51-producing *K. pneumoniae* clinical isolate 1B was the highest (2048 mg/L) in our study, while the CZA MIC for the KPC-51-harbouring *E. coli* DH5a transformant was only 8 mg/L. Other beta-lactamases detected in isolates from the same patient were identical.

**Table 1:** Characteristics of isolates recoverd from the same patients before (A) and after (B) ceftazidime/avibactam exposure

				MIC		Porin sequcence modifications	
			KPN clinical isolate		E.coli DH5a(clone)		
Strain	KPC^a^	β-lactamase	CZA^a^	CZA + 25 mg/ml PABN	CZA^a^	Ompk35^b^	Ompk36^c^
1A	KPC-2	TEM,SHV-64,CTX-M-65	2	^d^		truncated at 62 aa	GD insertion at 136–137 aa
3A	KPC-2	TEM,SHV-11,CTX-M-65	4			truncated at 62 aa	GD insertion at 136–137 aa
7A	KPC-2	SHV-64,OXA-10,DHA-1	4			truncated at 62 aa	GD insertion at 136–137 aa
8A	KPC-2	SHV-64,DHA-1	4	4	≤0.125	truncated at 62 aa	GD insertion at 136–137 aa
1B	KPC-51	TEM,SHV-64,CTX-M-65	2048	2048	8	truncated at 62 aa	GD insertion at 136–137 aa
3B	KPC-33,KPC-2	TEM,SHV-11,CTX-M-65	256	512	2	truncated at 62 aa	GD insertion at 136–137 aa
7B	KPC-52	SHV-64,OXA-10,DHA-1	256	512	32	truncated at 62 aa	GD insertion at 136–137 aa
8B	KPC-33	SHV-64,DHA-1	128	128	2	truncated at 62 aa	GD insertion at 136–137 aa

Notes

^a^ Data were from the reported paper [25].

^b^ Predicted translational modification of Ompk35 were based on the reference sequence (NCBI reference sequence WP_135730820.1) from *K. pneumoniae* ATCC 13883.

^c^ Predicted translational modification of Ompk36 were based on the reference sequence (GenBank accession number AEW62399.1) from *K. pneumoniae* ATCC 13883.

^d^ The MIC of CZA + 25 mg/mL PABN were not detected in isolates 1A,3A, and 7A.

Abbreviations: *CZA* ceftazidime/avibactam

### Outer membrane porin gene sequence analysis

Compared with the sequences of wild type *ompk35* (NCBI reference sequence WP_135730820.1) and *ompk36* (GenBank accession number AEW62399.1) genes from the reference strain *K. pneumoniae* ATCC13883, the *ompk35* sequence of all 8 isolates had a deletion after 85 bp, which caused a premature stop codon after amino acid position 62. And the Ompk36 in all 8 isolates had a glycine and aspartic acid duplication at amino acid 136 (136–137 GD insertion) (Table 1). Different sequences of Ompk35 and Ompk36 were not found between the baseline isolates (A) and CZA resistant isolates (B) recovered from the same patient.

### Functional restoration of OmpK35 and Ompk36

**Fig. 1:**
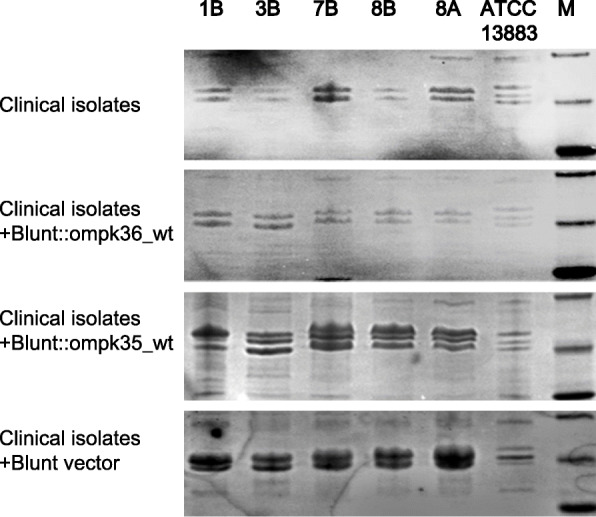
Outer membrane protein profiles of selected representative clinical isolates and corresponding transformants. M: protein markers of 45, 35 and 25 kDa. The bands showed in ATCC13883 were Ompk35, Ompk36 and OmpkA

The blunt vectors harboring functional wild-type Ompk35 or Ompk36 were transferred into selected isolates with different KPC variants by electroporation. The profiles of outer membrane proteins in all clinical isolates and transformants were analyzed by SDS-PAGE (Fig. 1). The truncated Ompk35 in clinical isolates were not shown, while the restoration of lost Ompk35 in corresponding transformants was confirmed in the profiles. The mutated Ompk36 did not influence the profiles of outer membrane proteins in clinical isolates. As a control, the empty blunt vector was also transferred into clinical isolates. The existence of wild type Ompk36 and empty blunt vector in corresponding transformants can not be reflected by SDS-PAGE, but it was confirmed using PCR and sequencing. Compared with clinical isolates with different KPC variants (8A, 1B, 3B, 7B, 8B), no significant reduction in CZA MICs was observed for selected isolates with the restoration of functional wild type Ompk35 or Ompk36 (Table 2). CZA MIC differences between clinical isolates, transformants with wild type Ompk35, transformants with wild type Ompk36, and transformants with original blunt vector were no more than 2-fold.

**Table 2:** Effects of restoration of wild type Ompk35 and Ompk36 into clinical KPC-KP isolates

Strain	Description	MIC (mg/L)					
		CZA	CAZ	IMP	MEM	ATM	FEP
8A	clinical isolate 8A	8	≥64	≥16	≥16	≥64	≥32
KPM30	8A + Blunt vector	4	≥64	≥16	≥16	≥64	≥32
KPM02	8A + Blunt::ompk35_wt	4	≥64	≥16	≥16	16	≥32
KPM06	8A + Blunt::ompk36_wt	8	≥64	≥16	≥16	≥64	≥32
1B	clinical isolate 1B	2048	≥64	1	4	≥64	≥32
KPM21	1B + Blunt vector	2048	≥64	0.5	4	≥64	≥32
KPM10	1B + Blunt::ompk35_wt	2048	≥64	1	≤0.25	≥64	≥32
KPM11	1B + Blunt::ompk36_wt	2048	≥64	0.5	0.5	≥64	≥32
3B	clinical isolate 3B	512	≥64	≥16	≥16	≥64	≥32
KPM23	3B + Blunt vector	512	≥64	≥16	≥16	≥64	≥32
KPM01	3B + Blunt::ompk35_wt	512	≥64	≥16	≥16	≥64	≥32
KPM05	3B + Blunt::ompk36_wt	512	≥64	≥16	≥16	≥64	≥32
7B	clinical isolate 7B	512	≥64	2	2	≥64	≥32
KPM27	7B + Blunt vector	512	≥64	2	2	≥64	≥32
KPM04	7B + Blunt::ompk35_wt	512	≥64	1	2	≥64	≥32
KPM08	7B + Blunt::ompk36_wt	512	≥64	1	2	≥64	≥32
8B	clinical isolate 8B	256	≥64	2	4	≥64	≥32
KPM28	8B + Blunt vector	256	≥64	1	4	≥64	≥32
KPM03	8B + Blunt::ompk35_wt	256	≥64	2	0.5	16	2
KPM07	8B + Blunt::ompk36_wt	256	≥64	1	4	≥64	≥32

Abbreviations: *CZA* ceftazidime/avibactam; *CAZ* ceftazidime; *IMP* imipenem; *MEM* meropenem; *ATM* aztreonam; *FEP* cefepime

### Gene expression and copy number of *bla*_KPC_, and *ompk35/36*

**Fig. 2:**
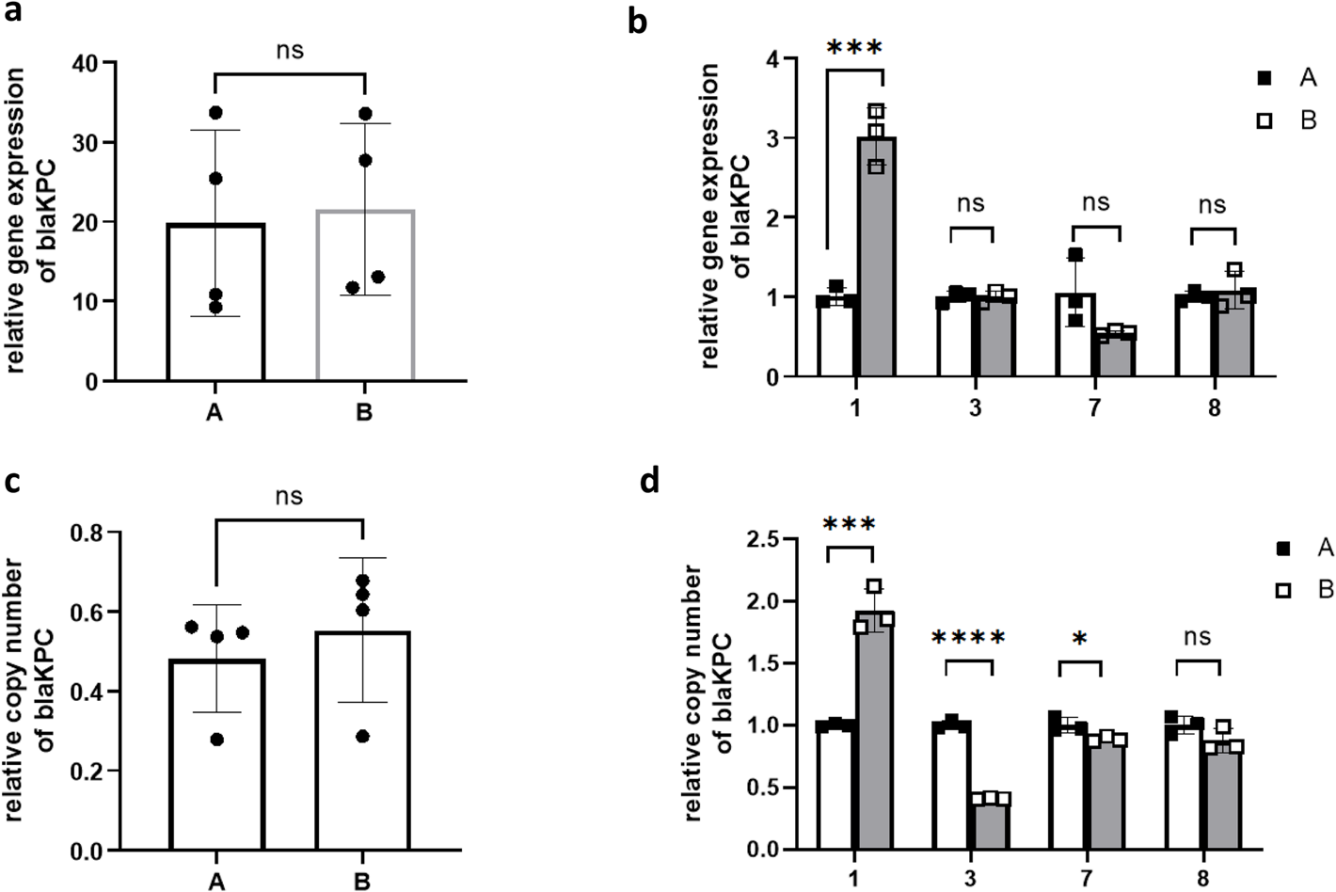
Relative gene expression (ab)and copy number (cd)of blaKPC in the baseline isolates (**A**) and CZA resistant isolates (**B**)

**Fig. 3:**
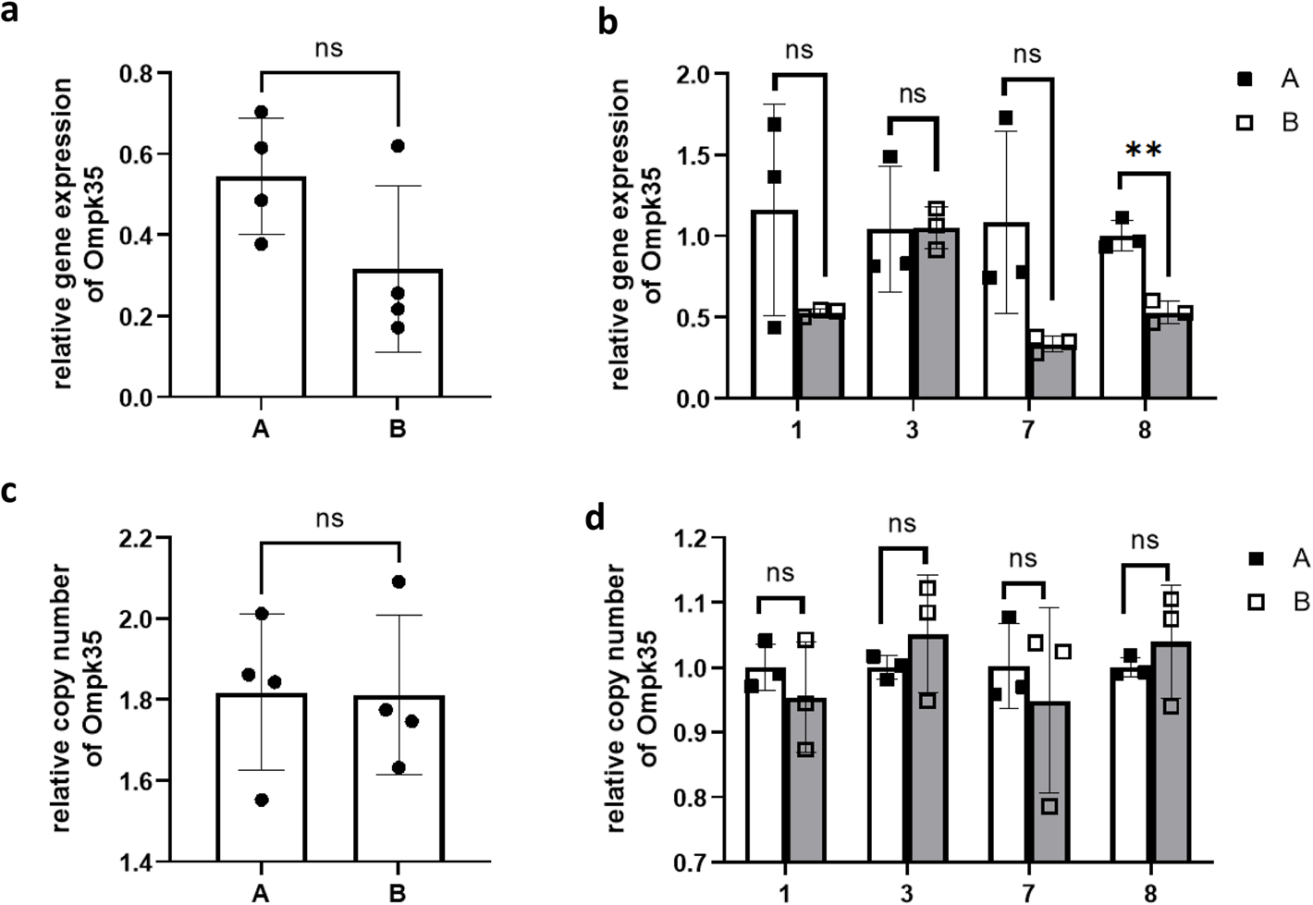
Relative gene expression (ab)and copy number (cd)of Ompk35 in the baseline isolates (**A**) and CZA resistant isolates (**B**)

**Fig. 4:**
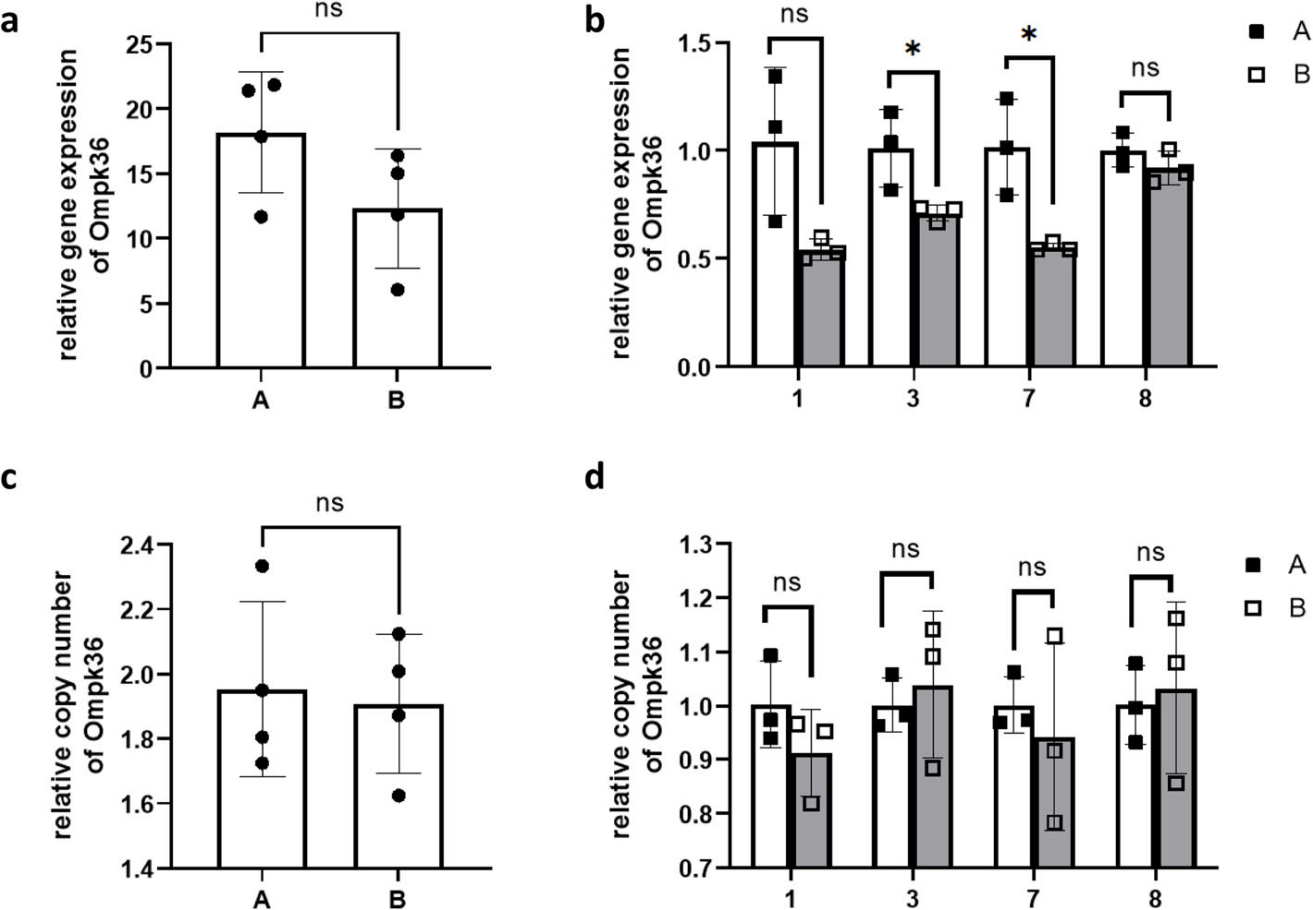
Relative gene expression (ab)and copy number (cd)of Ompk36 in the baseline isolates (**A**) and CZA resistant isolates (**B**)

There was no significant difference in relative gene expression and copy number of *bla*_KPC_*,* or *ompk35/36* between the baseline isolates (A) and CZA-resistant isolates (B) (Fig. 2a, c, Fig. 3a, c, Fig. 4a, c). When we compared the baseline isolate and the CZA-resistant isolate recovered from the same patient, the relative gene expression of *bla*_KPC_ in 1B was higher than 1A (Fig. 2b), while no significant difference was observed in isolates of other patients. The relative copy number of *bla*_KPC_ in 1B was higher than 1A, the relative copy number of *bla*_KPC_ in 3B and 7B were lower than 3A and 7A, and no significant difference in relative copy number was observed between 8A and 8B (Fig. 2d).

No significant difference of gene expression and copy number of *ompk35/36* were observed between 1A and 1B (Fig. 3b, d, Fig. 4b, d), while decreased *ompk35* gene expression was found in CZA-resistant isolate with *bla*_KPC-52_ (8B) (Fig. 3b), and decreased *ompk36* gene expression was found in other CZA-resistant isolates with *bla*_KPC-33_ (3B, and7B) (Fig. 4b).

### The role of the AcrAB efflux pump

After the addition of PABN, CZA MICs of all selected isolates were not decreased by more than 2-fold (Table 1). This indicated that efflux is not a major mechanism for resistance to CZA.

### Core genome phylogenetic analysis of isolated *K. pneumoniae*

**Fig. 5:**
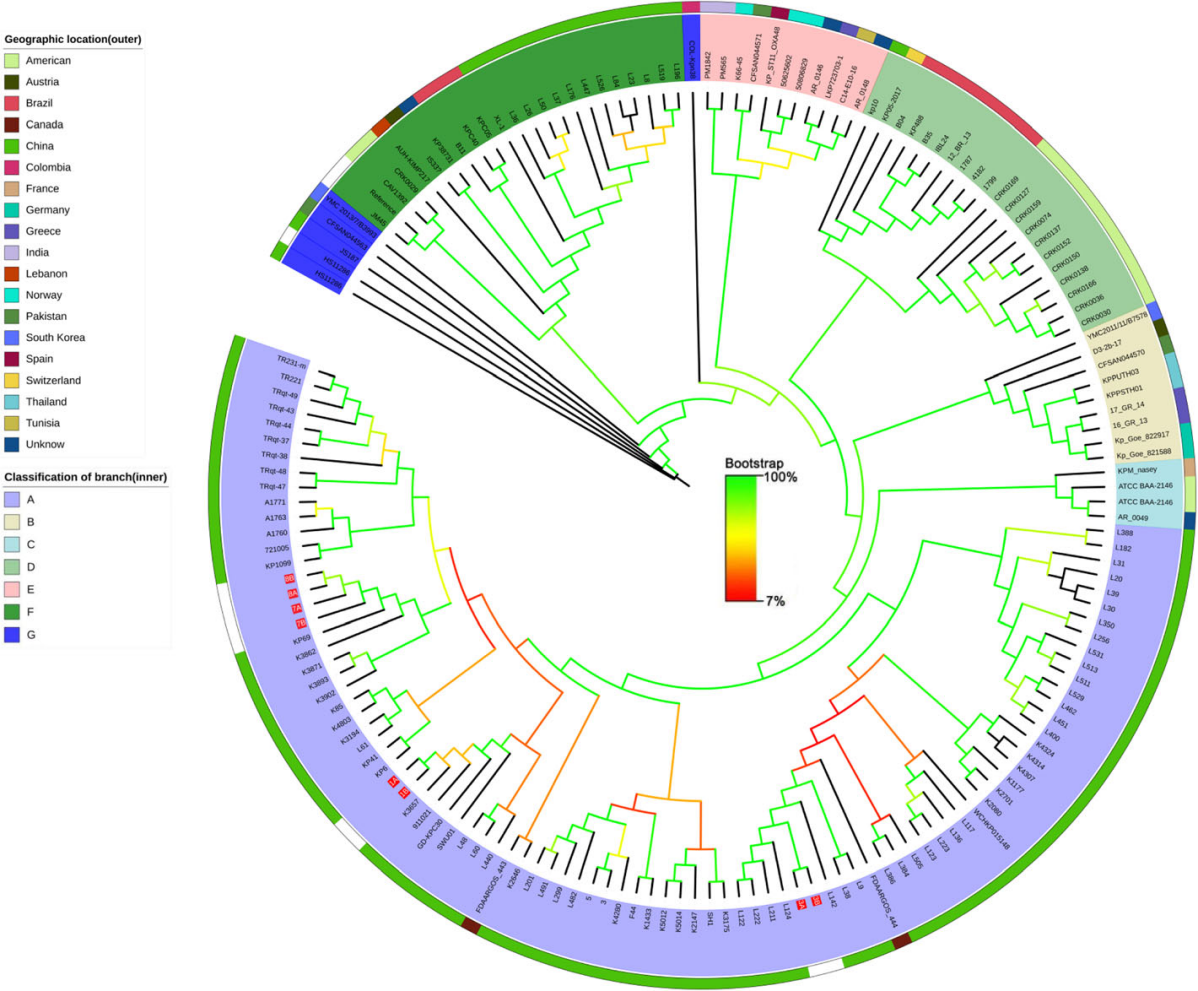
Maximum likelihood phylogeny based on SNPs in the core genomes of the *K. pneumoniae* ST11 strains isolated worldwide

Core genome phylogenetic analysis was performed to compare the 8 isolates from this study based on the results of WGS analysis and 157 of *K. pneumonia* strains filtered by MLST from NCBI (until July 2019). As shown in Fig. 5, all of the 8 isolates were located in a large branch (light purple, A-class) from the phylogenetic tree as a whole, which was almost from China except for 2 Canadian sources. Ten isolated strains can be divided into three groups. Groups 1 (isolate 3A, 3B) was the closest phylogenetic tree branch (L122, L222, L211, and L124) which were all isolated from Hangzhou China.

## Discussion

In our report, we demonstrate a new mechanism of high-level CZA resistance in a KPC-producing *K. pneumoniae* strain in a lung transplant recipient, which is that high-level resistance to CZA is due to increased gene expression and copy number of the mutated *bla*_KPC_. Though increased *bla*_KPC_ expression and copy number and/or Ompk defects were reported associated with increased CZA MICs [6, 21, 26], this result was often reported in isolates with wild type KPC, and the role of *bla*_KPC_ expression and porins in isolates with mutated *bla*_KPC_ is not clear. Our results have supplemented this evidence. In isolates with mutated *bla*_KPC_, the mutated *bla*_KPC_ may play a major role in CZA resistance, and the increased gene expression and copy number of the mutated *bla*_KPC_ could cause high-level CZA resistance, while the truncated Ompk35 may rarely contribute to the increased MIC of CZA.

Resistance to CZA has been reported in KPC-producing *K. pneumoniae* following treatment of CZA. Shields et al. first reported the evolution of CZA resistance during the treatment of *K. pneumoniae* infections in three patients from the United States in 2016 [3]. The treatment-emergent CZA resistance was subsequently described in Greece [27], Italy [9, 15, 16], Finland [10], Germany [12], and Spain [28] since then. And we recently reported four CZA-resistant KPC-KP recovered from lung transplant recipients after 13–22 days of CZA treatment in China [25].

KPC-31 [4] and KPC-33 [6] were the first two reported mechanisms associated with CZA resistance during treatment. Besides, KPC-35 [8], KPC-36 [15], KPC-41 [14], KPC-44 [10], KPC-48 [28], and KPC-57 [27] were also reported to be associated with acquired CZA resistance. We showed that CZA resistance in our 4 CZA-resistant isolates is due to mutations of *bla*_KPC_, and that two novel KPC variants (KPC-51, KPC-52) are also associated with CZA resistance [25]. The CZA MIC of the KPC-51-producing *K. pneumoniae* clinical isolate was the highest (2048 mg/L) in our study, while the CZA MIC for the KPC-51 *E. coli* DH5a transformant was only 8 mg/L. That indicated the variant KPC (KPC-51) is not the only mechanism of increased CZA MIC.

The role of mutated porins is not clear for CZA resistance. Modifications of Ompk35 and Ompk36, accompanied with various beta-lactamases, lead to carbapenem resistance, while do not influence CZA [29, 30]. However, several reports showed Ompk35/36 defects were associated with a high MIC of CZA. In the first reported CZA-resistant KPC-KP from a patient without CZA treatment [18], a nonfunctional Ompk35 (a premature stop codon at amino acid position 63) and a T333N mutation in Ompk36, were associated with impacted CZA susceptibility [22]. Castanheira et al. described a CZA-resistant *K. pneumoniae* displayed a premature stop codon in Ompk35 and decreased expression of Ompk36 [31]. Furthermore, Shields et al. reported mutations in Ompk36 were associated with CZA MICs [32]. Shen et al. showed that the restoration of functional Ompk35 resulted in a 2–4 fold decreased in the MICs of CZA for selected CZA-resistant isolates, indicating that the nonfunctional Ompk35 was related to CZA resistance [19]. Cui et al. reported the same *ompk35* and *ompk36* gene mutations were detected in reduced CZA susceptible strains and the CZA susceptible strains, which indicated that *ompk35/36* mutations only partially contribute to the reduced susceptibility of CZA in the study [33]. Similarly, we observed that a truncated Ompk35 and a GD insertion at amino acid position 136–137 in Ompk36 in all our isolates, including 4 CZA-resistant isolates and 4 CZA-susceptible isolates.

The reports of *K.pneumoniae* harboring mutated KPC combined with porins defects were rare. A novel KPC variant (KPC-36) and porins defects were discovered in a CZA-resistant *K.pneumoniae* ST1519 (MIC = 16 mg/L) [15]. In another CZA-resistant *K.pneumoniae* (MIC = 64 mg/L) harboring KPC-53, porins defects could be detected [34].

We hypothesize that the different KPC variants combined with Ompk35/36 defects could lead to the different levels of CZA MIC, and the KPC-51 coupled with porins defects may lead to the highest MIC of CZA. However, after the restoration of functional Ompk35 or Ompk36, no significantly decreased CZA MICs were observed in CZA-resistant isolates. We believed that the mutated *bla*_KPC_ was the most important mechanism in our isolates, and the mutations of Ompk35 or Ompk36 contribute no or rare to CZA resistance. The role of mutated porins for CZA should be further investigated.

The expression and copy number of *bla*_KPC_ were often associated with reduced susceptibility to CZA. Overexpression of the *bla*_KPC_ gene is a potential mechanism of CZA resistance in wild type *bla*_KPC_ isolates. Nelson et al. reported that porins alteration combined with increased *bla*_KPC-3_ gene copy number and gene expression can cause CZA resistance [22]. And the relative *bla*_KPC-2_ copy numbers and relative expression of *bla*_KPC-2_ in the reduced susceptibility group were significantly higher than those in the susceptibility group [19, 23, 33]. For mutated *bla*_KPC_, the report of KPC variants combined with gene expression and copy number is rare. As we writing this article, one study [34] showed the increased *bla*_KPC-53_ gene dosage (two copies) coupled with porins alterations may lead to high-level CZA resistance (MIC = 64 mg/L). But there was only one strain with increased *bla*_KPC-53_ gene dosage was reported in the study, and the comparison between isolates with different KPC variants was not investigated.

Our results showed that the relative gene expression and copy number of *bla*_KPC_ in isolate with the highest CZA MIC was higher than baseline isolate carrying wild type *bla*_KPC-2_, while this phenomenon did not appear in other CZA-resistant isolates. These results indicate that the mutated *bla*_KPC_ was the dominant mechanism of CZA resistance in our isolates, and when combined with increased gene expression and copy number of *bla*_KPC_ could lead to the higher level MIC of CZA.

This study is limited by its small patient population. CZA resistance was discovered before the widespread use of CZA in China. More research needs to be done, especially in data collected after the CZA treatment.

## Conclusions

In summary, we found that mutated *bla*_KPC_ is not the only mechanism related to CZA resistance, the increased gene expression and copy number of mutated *bla*_KPC_ can cause high-level CZA resistance. With the use of CZA, more CZA resistant isolates have been reported worldwide, and more mechanisms of CZA resistance need to be explored. In consideration of the rapid acquisition of CZA resistance after therapy, our founding suggests that it is crucial to monitor the MIC of CZA in KPC-KP.

## Methods

### Isolates

Eight previously described isolates [25], including 4 baseline isolates recovered before CZA treatment and 4 corresponding CZA-resistant isolates recovered after CZA treatment, were analyzed in the present study. *K. pneumoniae* ATCC13883, *K. pneumoniae* ATCC700603, *E.coli* ATCC25922, and *Salmonella enteric serotype Braenderup* H9812 were used as reference isolates.

### Antimicrobial susceptibility testing

Antimicrobial susceptibility testing performed using the VITEK-2 compact system (bioMerieux, Marcy-l’Etoile, France). Broth microdilution susceptibility testing of CZA was performed, and the result was interpreted according to the guidelines established by the Clinical and Laboratory Standards Institute (CLSI, M100, 2019). Avibactam was tested at a fixed concentration of 4 μg/ml in combination with increasing concentrations of ceftazidime. The reference strains *E. coli* ATCC25922, and *K. pneumoniae* ATCC700603 were used as controls.

### Detection of genes encoding β-lactamases, and outer membrane proteins

PCR detection for the presence of beta-lactamase genes encoding carbapenemases (*bla*_KPC_, *bla*_NDM-1_, *bla*_VIM_, *bla*_IMP_, and *bla*_OXA-48_), ESBL associated genes (*bla*_CTX-M_, *bla*_SHV_, and *bla*_TEM_), and plasmid-borne AmpC beta-lactamases (*bla*_ACC_, *bla*_DHA_, and *bla*_CMY_) were performed as described previously [35]. Outer membrane protein genes were amplified by PCR as described previously [36]. PCR amplicons were sequenced and compared with sequences available in the GenBank database using BLAST searches.

### Quantitative real-time PCR (qRT-PCR) determinating gene expression and copy number of *bla*_KPC_ and *ompk35/36*

The gene expression and copy number of *bla*_KPC_ and *ompk35/36* of 4 baseline isolates (A) and 4 CZA-resistant isolates (B) were examined as described previously [36–38], and the reference genes were listed in Table 3. Total RNA at mid-logarithmic growth phase bacterial cultures were obtained using RNeasy Mini Kit (Qiagen, Germany) and treated with RNase-Free DNase Set (Qiagen) in accordance with the manufacturer’s protocol. RT-PCR was performed using the QuantiTect SYBR Green RT-PCR Kit (Qiagen) on a QuantiStudio 12 K Flex system (Thermo Fisher Scientific). DNA was extracted by QIAamp DNA Mini Kit (Qiagen), and copy numbers were measured using the QuantiFast SYBR Green PCR Kit (Qiagen) on a QuantiStudio 12 K Flex system. *K. pneumoniae* house-keeping gene *rpoB* was used to normalize the gene expression and copy number of *bla*_KPC_ and *ompk35/36*. Statistical analyses were performed using GraphPad Prism 9.

**Table 3:** Primers used in this study

Genes	Sequence 5′-3′	Reference
KPC-F	GGCCGCCGTGCAATAC	[38]
KPC-R	GCCGCCCAACTCCTTCA	[38]
RPOB-F	CTGATGCCTCAGGATATGATCAAC	[38]
RPOB-R	CTGGCTGGAACCAAAGAACTCT	[38]
OmpK35-F	TCCCTGCCCTGCTGGTAG	[36]
OmpK35-R	CTGGTGTCGCCATTGGTGG	[36]
OmpK36-F	GCGACCAGACCTACATGCGT	[36]
OmpK36-R	AGTCGAAAGAGCCCGCGTC	[36]

### Cloning of *ompK36* and *ompK35* genes

The coding sequences of wild type *ompk35* and *ompk36* genes from reference isolate *K. pneumoniae* ATCC 13883 were amplified using primer pairs bluntOmpk35 (forward, 5′-AATGATGAAGCGCAATATTCT-3′; reverse, 5′- CGAAGGGGTGTACTGCAGATTA-3′) and bluntOmpk36 (forward, 5′- CATGAAAGTTAAAGTACTGTC-3′; reverse, 5′- TTATGCAGCTTGCAACTTAGAA-3′), respectively, and subsequently cloned into pEASY-Blunt vectors (Transgen Biotech Co., China). After nucleotide sequence verification, the kanamycin resistance gene of recombinant plasmids was replaced by the apramycin resistance gene using PCR. As a control, the kanamycin resistance gene in an empty vector was also replaced by the apramycin resistance gene. The recombinant plasmids were selected on Luria-Bertani (LB) agar containing apramycin at a concentration of 50 μg/ml.

### Preparation of competent cells and electroporation

The competent cells of *K. pneumoniae* clinical isolates were prepared using 10% glycerol as previously described [39]. The mixture of 50ul electrocompetent cells and 5ul plasmid was transferred into a 2 mm electroporation cuvette and electroporated using MicroPuler System (Bio-Rad) at 2.5 kV. The cells were plated onto Luria-Bertani (LB) agar containing apramycin at 50 mg/L. The plates were incubated at 37 °C overnight, and the successful clone was identified using PCR and sequencing. The MICs of the recombinant strains were determined in the presence of IPTG at 100 μM [22].

### Isolation and analysis of outer membrane components

Outer membrane proteins were isolated according to Carlone’s rapid procedure [40], and analyzed with SDS-PAGE.

### Efflux pump inhibitor tests

MICs of CZA in combination with PABN (phenylalanine-arginine beta-naphthylamide) (25 mg/mL) [19, 41], a pump inhibitor, were determined. A fourfold decrease in MIC after the addition of PABN was considered significant.

### Core genome phylogenetic analysis

WGS of all 8 isolates was carried out using the Illumina NovaSeq system in our previous study [25], and the assembled genome sequence has been deposited on NCBI with BioProject ‘PRJNA588110’. SNP analysis was performed using snippy software and the JM45 sequence was used for reference sequences. 8313 SNV sites were identified and the Modelfinder was used to find the best model. A phylogenetic tree was built by Iqtree [42, 43] with the best model (HKY + F + ASC + R4) and the number of bootstraps was set to 1000 times.

## Data Availability

Illumina sequence reads for the sequenced isolates in this study have been deposited in the NCBI sequence read archive are available in the sequence read archive under accessions ‘SRR10394536’(https://www.ncbi.nlm.nih.gov/search/all/?term=SRR10394536), ‘SRR10394605’(https://www.ncbi.nlm.nih.gov/search/all/?term=SRR10394605), ‘SRR10394910’(https://www.ncbi.nlm.nih.gov/search/all/?term=SRR10394910), ‘SRR10394933’(https://www.ncbi.nlm.nih.gov/search/all/?term=SRR10394933), ‘SRR10397948’(https://www.ncbi.nlm.nih.gov/search/all/?term=SRR10397948), ‘SRR10397950’(https://www.ncbi.nlm.nih.gov/search/all/?term=SRR10397950), ‘SRR10397951’(https://www.ncbi.nlm.nih.gov/search/all/?term=SRR10397951), ‘SRR10397953’(https://www.ncbi.nlm.nih.gov/search/all/?term=SRR10397953).

## Abbreviations

cIAI: complex intra-abdominal infections
CRE: Carbapenem-resistant enterobacerales
CRKP: Carbapenem-resistant *Klebsiella pneumonia*
CZA: Ceftazidime/avibactam
HAP: Hospital-acquired pneumonia
KPCs: *K. pneumoniae* carbapenemases
KPC-Kp: KPC-producing *K. pneumoniae*
LB: Luria-Bertani
MIC: Minimal inhibitory concentration
PABN: Phenylalanine-arginine beta-naphthylamide
qRT-PCR: Quantitative real-time PCR
ST11: Sequence type 11
